# A novel pathogenic *SLC12A5* missense variant in epilepsy of infancy with migrating focal seizures causes impaired KCC2 chloride extrusion

**DOI:** 10.3389/fnmol.2024.1372662

**Published:** 2024-04-10

**Authors:** Viivi Järvelä, Mira Hamze, Jonna Komulainen-Ebrahim, Elisa Rahikkala, Johanna Piispala, Mika Kallio, Salla M. Kangas, Tereza Nickl, Marko Huttula, Reetta Hinttala, Johanna Uusimaa, Igor Medina, Esa-Ville Immonen

**Affiliations:** ^1^Nano and Molecular Systems Research Unit, University of Oulu, Oulu, Finland; ^2^Research Unit of Clinical Medicine, University of Oulu, Oulu, Finland; ^3^Medical Research Center Oulu, University of Oulu and Oulu University Hospital, Oulu, Finland; ^4^INMED, INSERM, Aix-Marseille University, Marseille, France; ^5^Department of Children and Adolescents, Division of Pediatric Neurology, Oulu University Hospital, Oulu, Finland; ^6^Department of Clinical Genetics, Oulu University Hospital, Oulu, Finland; ^7^Department of Clinical Neurophysiology, Oulu University Hospital, Oulu, Finland; ^8^Laboratory of Transgenic Models of Diseases, Institute of Molecular Genetics of the Czech Academy of Sciences, Prague, Czechia; ^9^Biocenter Oulu, University of Oulu, Oulu, Finland

**Keywords:** *SLC12A5*, GABA, potassium-chloride co-transporter, chloride, Cl^−^, epilepsy, neurodevelopmental disorder

## Abstract

The potassium-chloride co-transporter 2, KCC2, is a neuron-specific ion transporter that plays a multifunctional role in neuronal development. In mature neurons, KCC2 maintains a low enough intracellular chloride concentration essential for inhibitory neurotransmission. During recent years, pathogenic variants in the KCC2 encoding gene *SLC12A5* affecting the functionality or expression of the transporter protein have been described in several patients with epilepsy of infancy with migrating focal seizures (EIMFS), a devastating early-onset developmental and epileptic encephalopathy. In this study, we identified a novel recessively inherited *SLC12A5* c.692G>A, p. (R231H) variant in a patient diagnosed with severe and drug-resistant EIMFS and profound intellectual disability. The functionality of the variant was assessed *in vitro* by means of gramicidin-perforated patch-clamp experiments and ammonium flux assay, both of which indicated a significant reduction in chloride extrusion. Based on surface immunolabeling, the variant showed a reduction in membrane expression. These findings implicate pathogenicity of the *SLC12A5* variant that leads to impaired inhibitory neurotransmission, increasing probability for hyperexcitability and epileptogenesis.

## 1 Introduction

The delicate balance of excitatory and inhibitory signaling in the central nervous system (CNS) is essential to its functionality and development. In the mature CNS, the main neurotransmitters responsible for fast inhibitory signaling are GABA (γ-aminobutyric acid) and glycine, which induce chloride (Cl^−^) currents mediated by type A GABA (GABA_A_R) receptors and glycine receptors (GlyR), respectively. Low enough intracellular chloride concentration ([Cl^−^]_in_) is essential for this Cl^−^-mediated signaling to be inhibitory as Cl^−^ flux through these receptor channels is passive. Therefore, the direction and strength of the Cl^−^ current is dictated by the electrochemical gradient of Cl^−^, which, in the cellular environment, is relatively sensitive to changes in [Cl^−^]_in_. Recorded in healthy mature neurons from adult (P30) acute rat (Tyzio et al., 2008) or mouse (Tyzio et al., 2014; Deidda et al., 2015) hippocampal slices, the reversal potential of GABA_A_R (E_GABA_) lies in the range of −65 to −80 mV, corresponding to [Cl^−^]_in_ being in the range between 4 and 8 mM. Similar [Cl^−^]_in_ values have been determined *in vivo* using ratiometric Cl^−^-sensitive probes in cortical neurons of adult mice (Sulis Sato et al., 2017; Boffi et al., 2018). Since this range of E_GABA_ lies close to the typical values of resting potentials of neurons, the activation of GABA_A_R does not introduce substantial Cl^−^ currents due to a low driving force for Cl^−^. However, the opened GABA_A_R channels still help to stabilize the membrane potential and reduce the excitability of neurons through shunting effects. If the activation of GABA_A_R occurs during neuronal depolarization, the driving force for GABA_A_R-mediated currents increases, leading to a hyperpolarizing influx of Cl^−^ that effectively raises the threshold for action potential firing. Accordingly, this inhibitory Cl^−^ influx would become compromised if [Cl^−^]_in_ were to increase significantly.

In mature neurons, low enough [Cl^−^]_in_ is largely maintained by the neuron specific potassium (K^+^) -chloride (Cl^−^) co-transporter type 2, KCC2, encoded by the gene *SLC12A5*, and in mature neurons KCC2 is thus integral for postsynaptic inhibitory signaling (Rivera et al., 1999; Hübner et al., 2001). This is due to its chloride extrusion capability, which derives its energy from the favorable electrochemical gradient of K^+^ and helps to maintain [Cl^−^]_in_ levels well below 10 mM. Furthermore, at these [Cl^−^]_in_ levels, the driving force for transporting K^+^ and Cl^−^ is relatively close to thermodynamic equilibrium. Since KCC2 co-transport is bidirectional, this also confers KCC2 the potential ability for K^+^ uptake during high neuronal activity (Payne, 1997), further underlying the importance of this co-transporter in neuronal function.

At the earlier stages of neuronal development before birth in rats and mice, the expression of KCC2 is low (Rivera et al., 1999; Stein et al., 2004), whereas the expression of Cl^−^importing Na^+^/K^+^/Cl^−^ co-transporter type 1 (NKCC1) is high, and thus, [Cl^−^]_in_ is reciprocally high (Achilles et al., 2007). Interestingly, this does not mean a reduced importance for KCC2, as in addition to the canonical Cl^−^ extrusion, KCC2 also has non-canonical functions that are of importance during neuronal maturation and play a part in, e.g., spine development and neuronal migration (Li et al., 2007; Horn et al., 2010). Due to the higher [Cl^−^]_in_, E_GABA_ and GlyR reversal potential (E_Gly_) are depolarized above neuronal resting potentials, leading to depolarizing ion fluxes (Ben-Ari, 2002). Especially in the case of GABA_A_R responses, this seems to have a major role in neuronal maturation (Kasyanov et al., 2004). During postnatal development, NKCC1 is progressively downregulated (Plotkin et al., 1997), while KCC2 is upregulated (Vanhatalo et al., 2005), resulting in a strengthened Cl^−^ extrusion and a reciprocal decrease in [Cl^−^]_in_. This leads to a shift in GABA_A_R responses from excitatory to inhibitory (Rivera et al., 1999), stabilizing the neural network activity. In the human neocortex, this transition takes place perinatally and reaches maturation at roughly 6 months after birth (Sedmak et al., 2016).

In recent years, several pathogenic variants of *SLC12A5* have been linked to, e.g., idiopathic generalized epilepsy (IGE), epilepsy of infancy with migrating focal seizures (EIMFS), autism spectrum disorder (ASD) and schizophrenia (SZ) (Kahle et al., 2014; Puskarjov et al., 2014; Merner et al., 2015; Stödberg et al., 2015; Saitsu et al., 2016; Saito et al., 2017), highlighting the importance of this protein in modulating neurodevelopment and neuronal inhibition and its potential as a therapeutic target for treating epilepsies (McMoneagle et al., 2023).

Here, we have reported in the KCC2 encoding gene *SLC12A5* a novel recessively inherited missense variant c.692G>A, p. (R231H) in a child whose parents are consanguineous. The child was homozygous for the variant, developed symptoms of EIMFS soon after birth, and suffered from frequent drug-resistant epileptic seizures and profound intellectual disability. The functionality of this *SLC12A5* variant was studied in *in vitro* heterologous expression models by using gramicidin-perforated patch-clamp and ammonium (${\text{NH}}_{4}^{+}$) flux assay. Both methods suggested a significantly impaired Cl^−^ extrusion capability for the variant. Furthermore, based on surface immunolabeling, it was found that the plasma membrane expression of the variant was also significantly decreased. These data strongly suggest the ion transport functionality to be compromised and hence pathogenicity for the identified *SLC12A5* variant, which could be the underlying cause for the severe epileptic encephalopathy of the patient.

## 2 Methods

### 2.1 Patient recruitment

Written informed consent was obtained from the parents of the patient. This study was conducted in accordance with the Declaration of Helsinki and was approved by the ethical review committee of Oulu University Hospital (EETTMK 33/2014, and amendment in 2021).

### 2.2 Electroencephalopgraphy

The patient's electroencephalogram (EEG) was recorded using a conventional commercial EEG machine, NicoletOne EEG System (Natus Medical). A modified set-up for neonatal recordings (excluding p9, t9, t10, p10 electrodes) with electrode placement according to the guidelines by the International Federation of Clinical Neurophysiology (IFCN) was used for collecting signals. The signals were amplified by the system's in-built 32-channel v32 amplifier, sampled at the rate of 500 Hz and bandpass-filtered using 0.053–500 Hz bandwidth.

### 2.3 Genetic testing and data analysis

Peripheral blood samples were collected and genomic DNA was extracted using an automated QIAsymphony device and the Qiagen Qiasymphony DSP DNA Midi Kit (Qiagen, Hilden, Germany). Whole genome sequencing was performed as part of clinical diagnostics in Centogene, Rostock, Germany. In brief, genomic DNA was fragmented by sonication and Illumina adapters were ligated to generated fragments for subsequent sequencing on the HiSeqX platform (Illumina) to yield an average coverage depth of 30 × . An end-to-end in-house bioinformatics pipeline including base calling, primary filtering of low-quality reads and probable artifacts, and annotation of variants was applied. All disease-causing variants reported in the Human Gene Mutation Database^®^ (HGMD), in ClinVar or in CentoMD^®^ (Trujillano et al., 2017) in addition to all variants with minor allele frequency (MAF) of < 1% in the population databases were considered. Evaluation was focused on coding exons along with flanking +/– intronic bases, but extended to the complete gene region for candidate genes or in search for a second previously described variant in autosomal recessive inheritance pattern. All pertinent inheritance patterns were considered. All identified variants were evaluated with respect to their pathogenicity and causality. Variants were categorized into five classes (pathogenic, likely pathogenic, variant of uncertain significance (VUS), likely benign, and benign) according to the American College of Medical Genetics (ACMG) guidelines (Richards et al., 2015).

Online *in silico* analysis tools were used to assess how deleterious the discovered c.692G>A, p. (R231H) substitution is predicted to be to the translated protein. These tools included the MutationTaster (Schwarz et al., 2014), Sorting Intolerant from Tolerant (SIFT, Sim et al., 2012), Polymorphism Phenotyping v2 (PolyPhen-2, Adzhubei et al., 2010), Rare Exome Variant Ensemble Learner (Revel, Ioannidis et al., 2016), Protein Variant Effect Analyzer (Provean, Choi and Chan, 2015), and Combined Annotation Dependent Depletion (CADD, Rentzsch et al., 2019).

### 2.4 Cell culture

Mouse neuroblastoma cells (N2a) were acquired from ATCC (ATCC; Neuro-2A CCL-131) and cultured on glass coverslips in either 35 mm dishes or in four well plates. Cells were incubated in 5% CO_2_ at 37°C in a 1:1 mixture of Dulbecco's modified Eagle's medium (Gibco) and Eagle's minimum essential medium (Sigma Aldrich) supplemented with 8%−10% FBS Good (PAN-Biotech), 2 mM GlutaMAX (Gibco), 100 I.U./ml penicillin and 100 μg/ml streptomycin (Cytiva HyClone). Cells up to a passage number of 10 were used for the perforated patch-clamp experiments, while passages up to 20 were used for the ${\text{NH}}_{4}^{+}$ flux assay and the immunolabelling.

### 2.5 Constructs

The vector inserts encoding the coding region of human KCC2b isoform (NM_020708.4; later denoted as KCC2) were synthetized by GenScript.com and subcloned into the previously described vectors harboring inserts of rat KCC2 (Kahle et al., 2014; Friedel et al., 2015). Namely, we used plasmid constructs with inserts of non-tagged KCC2 and KCC2 harboring an mCherry-tag linked to the N-terminus of the transporter, and KCC2 including an extracellular pHluorin-tag (KCC2-pH_ext_). The plasmid construct for the fluorescent protein mCherry pCAGImC_Empty was a gift from Joshua Mendell (Addgene plasmid #92015; http://n2t.net/addgene:92015; RRID:Addgene_92015; Golden et al., 2017). The single amino acid substitution of the identified variant c.692G>A (p.R231H) was introduced into the KCC2 constructs using QuikChange II site-directed mutagenesis kit (Agilent). The manufacturers protocol was followed. Bacterial transformations were conducted using stellar competent cells (Takara Bio; catalog number # 636763). The variant sites of the resulting plasmids KCC2^R231H^, mCherry-KCC2^R231H^ and KCC2^R231H^-pH_ext_ were sequenced by capillary sequencing to ensure a successful mutagenesis. Mutagenesis primers can be seen in Table 1, and sequencing primers in Table 2.

**Table 1:** Mutagenesis primers for generating the R231H-variant harboring plasmids encoding KCC2, mCherry -KCC2 and KCC2-pH_ext_.

**Plasmid**	**Primer sequence (5^′^>3^′^)**	**Direction**
KCC2	CATGCTGAACAACATGCATGTTTACGGCACCTGTG	Forward
KCC2	CACAGGTGCCGTAAACATGCATGTTGTTCAGCATG	Reverse
mCherry-KCC2	CATGCTGAACAACATGCATGTTTACGGCACCTGTG	Forward
mCherry-KCC2	CACAGGTGCCGTAAACATGCATGTTGTTCAGCATG	Reverse
KCC2-pH_ext_	GATGAACTTATTAATAACATGCATGTTTACGGCACCTGTGTG	Forward
KCC2-pH_ext_	CACACAGGTGCCGTAAACATGCATGTTATTAATAAGTTCATC	Reverse

**Table 2:** Primers for sequencing the variant sites of the generated KCC2-encoding plasmids harboring the R231H-variant.

**Plasmid**	**Primer sequence (5^′^> 3^′^)**	**Direction**
KCC2^R231H^, mCherry-KCC2^R231H^	TTCAAGGCAGAAGATGCCAGT	Forward
KCC2^R231H^, mCherry-KCC2^R231H^	GGGTACCATCGAAATCCTGCT	Forward
KCC2^R231H^, mCherry-KCC2^R231H^	ATGGAGAGGATGACACAACCC	Reverse
KCC2^R231H^-pH_ext_	TCCCAACGAAAAGAGAGACCA	Forward
KCC2^R231H^-pH_ext_	CCAGCATAGATGGCCAGGAT	Reverse

### 2.6 Heterologous expression

*In vitro* heterologous expression was performed by transiently transfecting N2a cells using either Lipofectamine 3000 (Invitrogen, Thermo Fisher Scientific) or a combination of Lipofectamine 2000 (Invitrogen, Thermo Fisher Scientific) and CombiMag Magnetofection Transfection Reagent (OZ Biosciences) according to manufacturers' protocols.

During transfections with Lipofectamine 3000, cells were incubated with the transfection reactions at 37°C in 5% CO_2_ in Opti-MEM (Gibco) supplemented only with 7% FBS to optimize transfection efficiency and cell health. Media was exchanged for the supplemented DMEM-EMEM after 24 h of incubation with the transfection reactions. Transfected cells were used for the gramicidin-perforated patch-clamp experiments 48–72 h after transfections.

During transfections with Lipofectamine 2000 and CombiMag Transfection Reagent, cells were incubated in the supplemented DMEM-EMEM media with the transfection reactions on a magnetic plate for 3h at 37°C and in 5% CO_2_. After 3 h, the plates were moved off the magnetic plate and media was exchanged for fresh supplemented DMEM-EMEM. Transfected cells were used for either ${\text{NH}}_{4}^{+}$ flux experiments or immunolabeling experiments 48–72 h after transfections.

### 2.7 Gramicidin perforated patch-clamp experiments

N2a cells cultured on glass coverslips were transiently co-transfected with the α1 subunit of glycine receptor (α1-GlyR) and either wild-type mCherry-KCC2, mCherry-KCC2^R231H^ or the fluorescent protein mCherry (mock) using Lipofectamine 3000 (cDNA ratio 2:1). The coverslips were submerged in a recording chamber and perfused with an extracellular solution containing (in mM) 140 NaCl, 2.5 KCl, 20 Hepes, 20 D-glucose, 2.0 CaCl_2_, 2.0 MgCl_2_, and 0.02 bumetanide, which is a loop diuretic that inhibits NKCC's and KCC's with a greater affinity for NKCC's (Payne et al., 2003). Here, bumetanide was used in low concentrations to block NKCC1 expressed in N2a cells and to prevent compensatory Cl^−^-dependent changes of NKCC1 activity and respective NKCC1-mediated flux of Na^+^, K^+^, and Cl^−^ ions (Gillen and Forbush, 1999). pH of the extracellular solution was adjusted to 7.4 with NaOH and the osmolality was measured to vary in the range of 305–315 mOsm. The cells were visualized under an upright microscope (Nikon Eclipse FN1) with an epifluorescence system and wild-type KCC2 (KCC2^WT^)-/KCC2^R231H^-/mock-transfected cells were identified by fluorescence. Recording micropipettes (3–6 MΩ) were manufactured from borosilicate capillaries (G150F-4, Harvard Apparatus or GC150F-10, Clark by Warner Instruments) with the P-87 Flaming/Brown horizontal Micropipette Puller (Sutter Instrument). The pipette solution contained (in mM) 150 KCl and 10 Hepes, and the pH of the pipette solution was adjusted to 7.2 with KOH. Gramicidin stock solution was prepared by dissolving gramicidin D (Sigma Aldrich) in DMSO to a concentration of 20 mg/ml, and gramicidin-containing pipette solution was prepared by adding gramicidin to the pipette solution to a final concentration of 10–20 μg/ml. The osmolality of the intrapipette solution varied in the range of 285–295 mOsm. The osmolality difference between the intrapipette and extracellular solutions was kept in the range of 10–20 mOsm to ensure optimal conditions for gigaseal formation. Glycine stock solution was prepared by dissolving glycine (Sigma Aldrich) in dH_2_O to a concentration of 100 mM, and a final concentration of 50–100 μM in the extracellular solution was used for the recordings. Glycine was applied focally and transiently to the cells with a recording micropipette connected to a Picospritzer (Harvard Apparatus, 5 p.s.i.). The micropipettes were controlled with micromanipulators (Sensapex).

After a successful formation of a gigaseal, a few minutes were waited to give the gramicidin time to perforate the cell membrane. The presence of glycine receptors was tested by focally applying glycine in the vicinity of the cell, and if glycine receptor mediated currents could be detected, recordings were commenced once the access resistance (*R*_a_) had lowered to < 150 MΩ. The membrane resistance (*R*_m_), resting potential (*V*_r_), cell capacitance (*C*_m_) and *R*_a_ were noted. To determine the E_Gly_, a voltage-clamp protocol was recorded. The protocol consisted of voltage steps with 10 mV increments, during which a 50 ms glycine pulse was applied focally to the recorded cell. Between glycine pulses, 10 s were waited for Cl^−^ clearance. The voltage steps were adjusted to induce several inward and outward GlyR current responses around the E_Gly_ in each cell. The holding potential was set to −60 mV. The current amplitudes were kept within a desired range so that the signal-to-noise ratio (SNR) was acceptable, but the largest current amplitudes were in the range of < 400 picoamperes to avoid extensive chloride build-up and to minimize voltage drops due to uncompensated series resistance. This was done by adjusting the positioning of the Picospritzer pipette. Pipette capacitance was compensated for, but cell capacitance was not. However, compensating for the cell capacitance was not meaningful in terms of voltage-clamp speed as glycine pulses were applied *t* = 500 ms into the voltage steps, well after charging transients that could interfere with the glycine currents. Voltage drops were also determined to be very small for the recorded GlyR currents. Liquid junction potential was determined to be ~4.5 mV and was accounted for in analysis.

Recordings were performed with Multiclamp 700B amplifier (Molecular Devices), digitized using Axon Digidata 1550B data acquisition system (Molecular Devices) and data collected using pCLAMP 10.7 acquisition software (Molecular Devices). All recordings were sampled at 10 kHz and filtered using the 4-pole Bessel filter of the amplifier at the cutoff frequency of 2 kHz. Data analysis was performed with a combination of Clampfit 10.7.0.3 (Molecular Devices), Microsoft Excel and Origin Pro 2020b (Origin Lab). Only cells with *R*_a_ < 150 MΩ and *R*_m_ > 400 MΩ were included in the analysis.

### 2.8 ${\text{NH}}_{4}^{+}$ flux assay

N2a cells were transiently co-transfected with the pH-sensitive protein pHluorin (Miesenböck et al., 1998) and either WT mCherry-KCC2, mCherry-KCC2^R231H^ or mCherry (mock) using Lipofectamine 2000 and CombiMag (pHluorin:KCC2 ratio 1:40). When applying such transfection conditions, all cells expressing pHluorin also expressed mCherry-KCC2 or mCherry. For recordings, coverslips were submerged in a recording chamber and perfused with an extracellular solution containing (in mM) 140 NaCl, 2.5 KCl, 20 Hepes, 20 D-glucose, 2.0 CaCl_2_, 2.0 MgCl_2_, and 0.02 bumetanide, pH 7.4. The ratiometric fluorescence of pHluorine/mCherry-KCC2 was measured using an epifluorescence imaging setup mounted on an inverted Olympus microscope (IX71, Olympus, Rungis, France) equipped with a FITC/CY3 Dualband ET Filterset (59009) and additional single-band excitation and emission filters included in two filter wheels (Lambda 10-B, Sutter Instruments Company, Novato, USA). The pH-sensitive fluorescence of pHluorine (F_480_) was obtained using fluorophore excitation with 480/20 filter (ET480/20) and 520/40 emission filter (ET520/40m). The fluorescence of pH-insensitive mCherry-KCC2 (F_577_) was obtained using 577/25 excitation filter (ET577/25x) and 645/75 emission filter (ET645/75m). All filters and filter sets were from Chroma.com. The fluorescence signal was sampled at 0.1 Hz using a CoolSNAPHQ Monochrome CCD camera and MetaMorph software (Molecular Devices Corp). Excitation lasted 100 ms for both wavelengths. All recordings were performed using a LUCPlanFLN 20 × objective (NA 0.45), allowing simultaneous recording of 5–10 transfected cells.

A baseline fluorescence signal was acquired for *T* = 5 min. Thereafter, the cells were perfused for 6 min with an extracellular solution containing 10 mM NH_4_Cl. A washout was performed after perfusion with the ${\text{NH}}_{4}^{+}$ containing solution and the cells were imaged for an additional 2–5 min during washout.

The images of pHluorine were converted off-line in Δ*F*/*F* = [*F*(*t*) – *F*0)/*F*0 ratio, where *F*(*t*) is the *F*_480_ value at a given time and *F*0 is the mean resting *F*_480_ value calculated during 2 min prior to application of ${\text{NH}}_{4}^{+}$ containing solution. The acidification rate (ΔR/min) was determined as the difference between ΔF/F values measured at 0.5 and 5 min of each recording in the presence of ${\text{NH}}_{4}^{+}$.

### 2.9 Surface immunolabeling

N2a cells were transiently transfected with either of two KCC2 constructs harboring an external pHluorin-tag, KCC2^WT^-pH_ext_ or KCC2^R231H^-pH_ext_, using Lipofectamine 2000 in combination with CombiMag Transfection Reagent according to the manufacturer's protocol. A separate set of experiments included N2a cells transfected with rat KCC2^WT^-pH_ext_ (rKCC2^WT^-pH_ext_) and ΔN-rKCC2-pH_ext_, a previously described construct (Friedel et al., 2017) with the N-terminus deleted and thus serving as a negative control due to its inability to attend the cell surface. Polyclonal rabbit anti-GFP antibody (SAB4701015, Sigma-Aldrich) used to reveal the pHluorin-tag was diluted in culture media (1:250). The cells were incubated with the antibody containing media at 37°C in 5% CO_2_ for 1 h and washed three times with fresh culture media after incubation. Next, the cells were placed into a thermos-isolated box at 13°C and incubated with the anti-rabbit Alexa 647 conjugated antibody (1:250, A-21244, Invitrogen) for 30 min. The antibody was diluted with Hepes-buffered saline solution (containing, in mM: 140 NaCl, 2.5 KCl, 20 Hepes, 20 D-glucose, 2.0 CaCl_2_, 2.0 MgCl_2_). This procedure allowed decoration of surface located KCC2-pH_ext_ at the moment of cooling down of the cells (*F*_m_, membrane fluorescence pool). After incubation, the cells were carefully washed with 13°C Hepes-buffered saline twice and fixed by incubating in PFA at 4°C for 30 min. The fixed cells were washed with PBS at room temperature (RT) and incubated for 60 min at RT in a mixture of 0.3 M glycine, 0.3% Triton X-100 and 5% goat serum (GS) to permeabilize the plasma membrane and block non-specific antibody binding. The fixed and permeabilized cells were incubated at RT with goat anti-rabbit Alexa 555-conjugated antibody (1:400, A-21428, Invitrogen) to reveal the internalized molecules labeled above using anti-GFP antibody at 37°C in 5% CO_2_ for 1 h. Then, the cells were stained with chicken anti-GFP antibody (1:500, 1020, Aves labs) followed with staining using Alexa Fluor™ 488 goat anti-chicken antibody (1:1,000, A-11039, Invitrogen) to reveal the total amount of KCC2-pH_ext_ overexpressed into the cells (*F*_t_). The amount of internalized KCC2-pH_ext_ (*F*_i_, internalized fluorescence pool) was quantified *post-hoc* using MetaMorph software (Molecular Devices) and included only Alexa 555, but not Alexa 647 positive molecules. All solutions containing diluted primary or secondary antibodies were cleared by centrifugation at 8,000 g for 5 min.

For quantitative analysis, images of labeled cells were acquired with an Olympus Fluorview-500 confocal microscope with a 60 × oil-immersion objective (NA 1.4), zoom 3 and 15 pixels/μm image resolution. Transfected cells were randomly selected based on Alexa Fluor 488 fluorescence only, followed by acquirement of z-stack images of Alexa Fluor 488, Alexa Fluor 555 and Alexa Fluor 647 fluorochrome-emitted fluorescence using green (excitation 488 nm, emission 505–525 nm), red (excitation 543 nm, emission 560–600 nm), and infrared (excitation 633, emission 660 nm) channels of the microscope, respectively. Each *z*-stack included 12 planes of 1 μm optical thickness and 0.7 μm distance between planes. The fluorescence intensities of labeled KCC2-pH_ext_ of each cell were collected with MetaMorph software. For statistical analysis of *F*_i_, *F*_m_, and *F*_t_ we used absolute fluorescence values and normalized each of them by their respective KCC2^WT^-pH_ext_ mean values to improve data visualization. The same principle was also used for calculating the *F*_i_/*F*_m_ ratios.

### 2.10 Statistics

Statistical analyses were performed using either Microsoft Excel or OriginPro 2020b (OriginLab). No blinding was performed for any of the experiments. Due to either small sample sizes or non-gaussian data distribution (tested using the Shapiro–Wilk normality test with a significance level of *p* = 0.05), non-parametric statistics were used. To test statistically significant differences between several groups, Kruskal–Wallis analysis of variance on ranks was performed, followed by Dunn's *post hoc* test for pairwise comparisons. In two-group paired comparisons, the Mann–Whitney U test was used. In all tests, the significance level was set to *p* = 0.05. All data are presented as medians with 25–75 interquartile ranges in parentheses, unless stated otherwise. Box plots with whiskers presenting data minima and maxima were used for visual presentation of data. In the figure legends, *N* represents the number of experiments and *n* represents the number of cells for each condition.

### 2.11 Data availability

The original datasets for the gramicidin-perforated patch-clamp (Järvelä and Immonen, 2024), ${\text{NH}}_{4}^{+}$ flux assay (Järvelä et al., 2024a) and immunolabelling (Järvelä et al., 2024b) are available in the publicly accessible IDA repository (www.fairdata.fi).

The datasets generated during genetic testing and whole-genome sequencing may reveal enough variants to identify an individual and are therefore not publicly available due to confidentiality and privacy reasons. For well-justified reasons, the data related to clinical analyses may be requested from the corresponding authors.

## 3 Results

### 3.1 Clinical report

**Figure 1:**
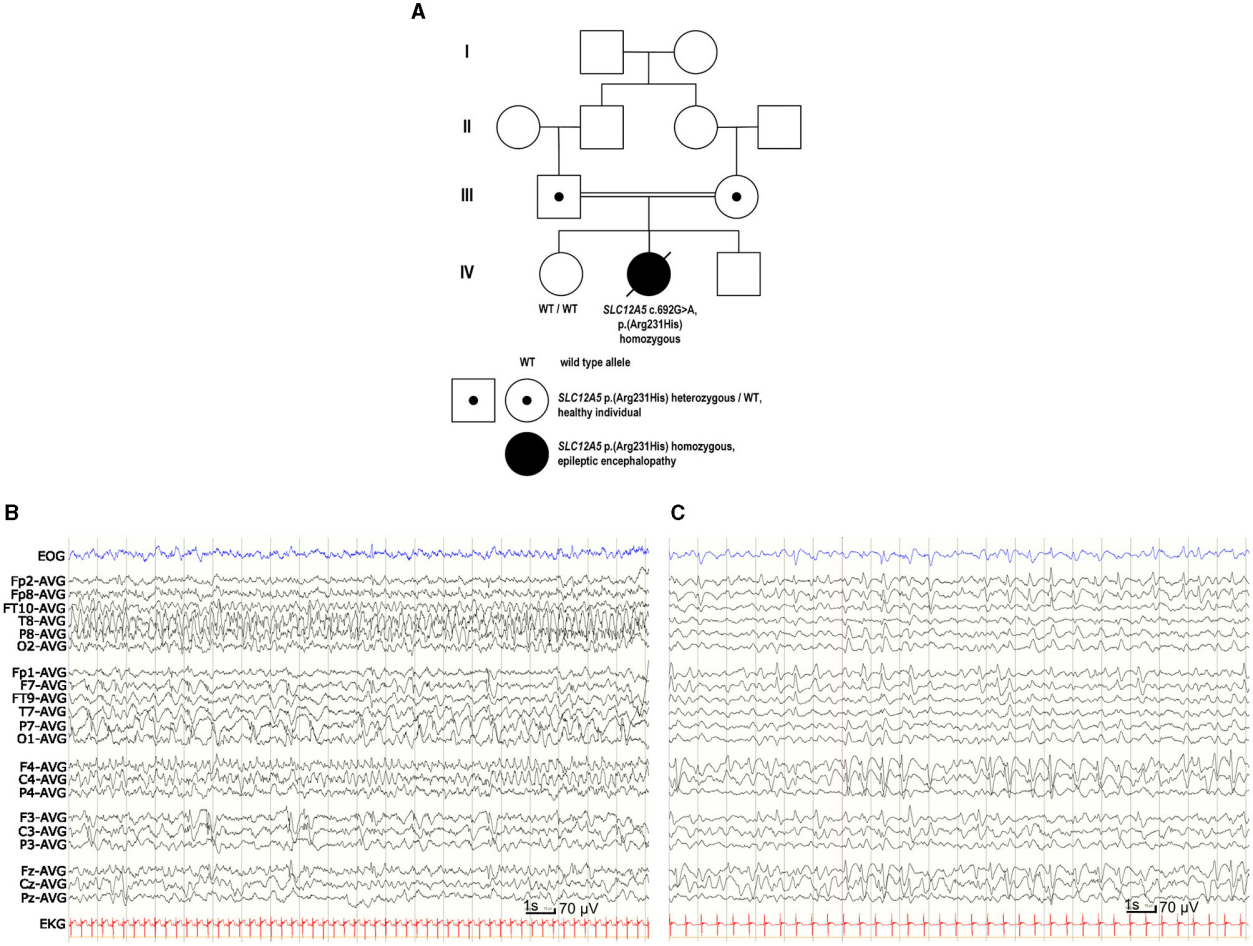
Family pedigree and EEG of the patient with EIMFS. **(A)** Four-generation family pedigree showing the recessive segregation of the *SLC12A5* c.692G>A p. (R231H) variant. Healthy older sister (IV-1) did not carry the *SLC12A5* c.692G>A p. (R231H) variant. **(B)** EEG at the age of 1 month showed seizure activity for more than 50% of the recording, spreading from posterior right hemisphere to the frontal areas and migrating to the left hemisphere. **(C)** At the age of 2 years and 5 months the background EEG was mainly slow delta activity. There was nearly continuous slow spike-and-wave- activity predominantly on the right side and one clinically evident seizure with head and eye deviation. During sleep there were multiple inactive 3–6 s periods without any visible symptoms.

Patient (Figure 1A, IV-2) was the second child of consanguineous Finnish parents. She was born at gestational weeks 41 + 3 after an uncomplicated pregnancy. Her birth weight was 4,315 g, height 53 cm (1.5 SD), occipitofrontal head circumference (OFC) 38 cm (2.5 SD) and the Apgar scores at 1/5/10 min were 8/9/9.

Her epilepsy fulfilled the criteria for EIMFS (Zuberi et al., 2022). Soon after birth, she started having seizures during which she was stiffening her body and her eyes were deviating, and she was monitored at the neonatal intensive care unit (ICU). She developed multiple seizure symptoms, including apneas, bending her head backwards, tonic posturing of her limbs, and twitching in her upper or lower limbs. During the years she had almost continuous focal migrating seizure activity, during which her eyes would deviate with clonic jerks and there would be clonia of the mouth corner and head version to the same side. Sometimes clonic jerks would also be seen on the upper limbs at the same side. During the same seizure the semiology would change sides. She also had focal tonic-clonic seizures starting with a scream, stiffening and apnea and continuing with clonic jerks lasting for minutes.

Her epilepsy was refractory to antiepileptic medications. After birth, she was treated in the ICU with phenytoin, phenobarbital, midazolam, ketamine, sodium thiopental, levetiracetam, topiramate, lacosamide, and lidocaine with poor response. She also received pyridoxine, pyridoxal phosphate and calcium folinate treatments without response. Ketogenic diet was started at the age of 1 month and discontinued at the age of 3.5 months due to lack of treatment response, and levetiracetam and sodium valproate were continued as antiepileptics. Due to poor seizure control, potassium bromide medication was started in addition to levetiracetam and sodium valproate medications at the age of 2 years. At that time, she had more than 10 focal tonic-clonic seizures daily. The potassium bromide medication reduced the frequency of focal tonic-clonic seizures to few a day with the dose of 40 mg/kg/day, serum level 15–20 mmol/L.

She has also had several fractures. At the age of 1 year and 8 months she had a distal femur fracture in her right leg, and at the age of 1 year and 9 months she had fractures in her left tibia and fibula and left femur. There were no known traumas preceding the fractures and it was assessed that the fractures had occurred during seizures. Furthermore, compression fractures have been identified in her vertebrae. Bone X-ray revealed osteopenia treated with vitamin D, calcium, bisphosphonate and regular physiotherapy. She has also presented with recurrent pneumonias leading to hospitalizations. She died due to pneumonia at the age of 4 years and 5 months. Autopsy revealed acute neutrophil infiltrates, edema, and partly fibrosis in the pulmonary alveoli as well as pleural fluid confirming the clinical diagnosis of pneumonia. Due to sepsis, she also had 80 ml of pericardial fluid. Autopsy of her brain revealed agenesis of corpus callosum, markedly enlarged lateral ventricles and scarcity of white matter in her brain. In addition, she had cholangitis and cholestasis which were estimated to be side effects of sodium valproate medication.

In the psychological assessment (Bayley-III) at the age of 2 years her developmental age corresponded to 2 months consistent with profound intellectual disability. She slept most of the time during the day and when she was awake, she tended to keep her eyes closed. She reacted to familial sounds by smiling and trying to turn her head and make eye contact.

In the clinical examination at 2 years, her height was 84.5 cm (−1.2 SD), weight 11.2 kg, and OFC 46.4 (−1.7 SD). She was hypotonic with intermittent hypertonia during seizures. She was not able to roll over or grasp objects. She also had facial hypotonia with an open-mouth appearance and a tented upper lip vermilion. She could vocalize, and she was drooling a lot. She had nystagmus in her eyes, and intermittent eye contact could be obtained.

### 3.2 EEG and brain magnetic resonance imaging

Continuous video-EEG monitoring was started at the age of 1 day and continued for 3 weeks. Interictal EEG revealed immature background activity and excessive multifocal sharp transients. Ictal EEG showed discharges lasting from 2 min to over 20 min, appearing four times in an hour and sometimes nearly continuously. Epileptic discharges migrated from one lobe or hemisphere to the other side. During the epileptic discharges there was twitching in her upper body but often no visible seizure signs. Similar focal migrating discharges were seen at the age of 1, 3, and 4 months (Figure 1B). From the age of 7 months the EEG showed mostly slower spike and wave discharges. Although the effect of potassium bromide was clinically clear, possibly due to high permeability of GABA_A_R to bromide (surrogate for Cl^−^; Suzuki et al., 1994), the EEG remained similar at the age of 2 years 5 months (Figure 1C).

After birth, magnetic resonance imaging (MRI) of her brain and spinal cord as well as the ultrasound of her heart were normal. Metabolic investigations including urine organic acids, plasma amino acids, serum very long chain amino acids, liver function tests, and NH_4_ were normal. Cerebrospinal fluid (CSF) glycine, lactate, pyruvate, neurotransmitters, Herpes simplex virus DNA and cytomegalovirus DNA analysis were negative. CSF/blood glucose ratio was normal. Array-comparative genomic hybridization (array-CGH) using HumanCytoSNP-12 v.2.1 Illumina showed a normal karyotype.

### 3.3 Whole genome sequencing and *in silico* analysis of the variant

*SLC12A5* (NM_020708.5) c.692G>A p. (R231H) variant (GRCh38 g.20:46040452G>A, rs1555863134) in exon 7 was identified in a homozygous state in the whole genome sequencing. No other variants were reported. Parents were healthy heterozygous carriers of this variant, and her healthy older sister did not carry this variant (Figure 1A). In the Genome Aggregation Database (gnomAD v.3.1.1, accessed on 8th October 2023) there were no *SLC12A5* c.692G>A p. (R231H) homozygotes, but there was one heterozygous carrier of this variant resulting in a minor allele frequency (MAF) of 0.00000657. *In silico* analysis predicted this variant as probably damaging to protein function or structure (Mutation Taster: deleterious, SIFT: pathogenic supporting with score 0, PolyPhen-2: probably damaging with score 1, Revel: pathogenic moderate with score 0.931, Provean: pathogenic supporting with score −4.9), and its CADD score was 32. The variant had been reported three times in Clinvar (variation ID 452646) and classified as a variant of unknown significance. Based on the results of our functional analysis and clinical data, we re-classified this variant as pathogenic according to the ACMG guidelines (PS3 based on the functional studies supporting pathogenicity, PM2 based on very low MAF in the gnomAD including no homozygotes, PP2 based on a missense variant in a gene that has a low rate of benign missense variation and in which missense variants are a common mechanism of disease, PP3 based on multiple lines of computational evidence supporting deleterious effect on the gene product, PP4 based on patient's EIMFS phenotype highly specific for *SLC12A5*-related epileptic encephalopathy).

### 3.4 Functionality of the variant

To find out whether the variant affects the Cl^−^ extrusion capability of the KCC2 protein, the functionality of KCC2^R231H^ was studied in *in vitro* heterologous expression models with two different approaches, gramicidin-perforated patch-clamp and ${\text{NH}}_{4}^{+}$ flux assay. The gramicidin-perforated patch-clamp recordings allow determining the E_Gly_, i.e., [Cl^−^]_in_, as a function of KCC2 activity, while the ${\text{NH}}_{4}^{+}$ flux assay provides the estimation of the ion-transport efficacy of the transporter (Medina and Pisella, 2020). For both methods, heterologous expression in N2a cells utilizing the KCC2b splice variant instead of KCC2a was opted for, as KCC2b is the primary isoform in mature neurons, undergoing up to a 20-fold increase in expression in mice during cortical development as opposed to a two-fold increase for KCC2a (Uvarov et al., 2009; Markkanen et al., 2014). Thus, KCC2b is primarily responsible for Cl^−^ extrusion in the mature neuron.

**Figure 2:**
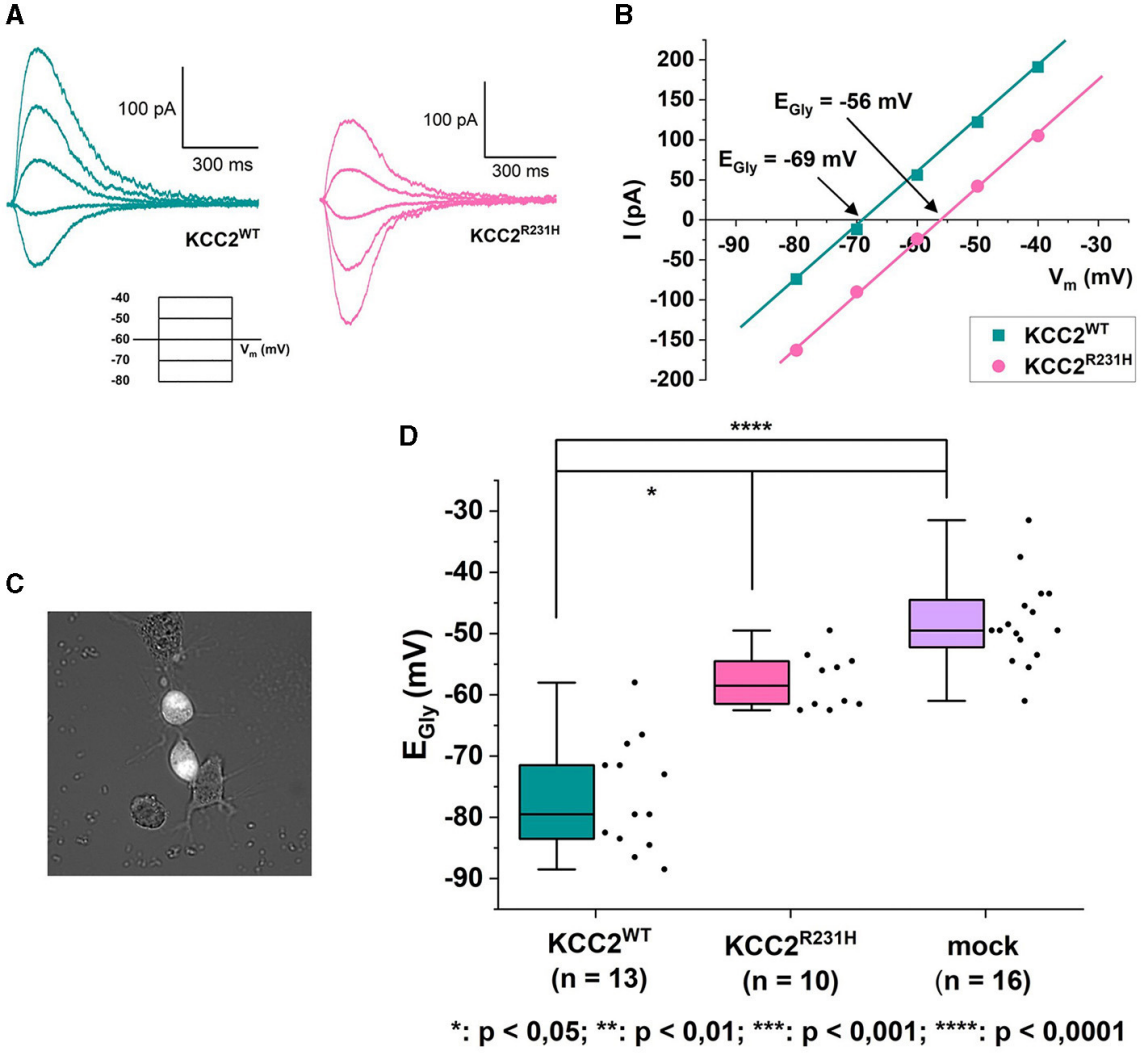
Gramicidin-perforated patch-clamp on N2a cells transiently co-transfected with KCC2^WT^ or KCC2^R231H^ and α1-GlyR. **(A)** Example traces of GlyR currents recorded in voltage-clamp mode from N2a cells expressing KCC2^WT^ and KCC2^R231H^. For clarity, only traces recorded under identical clamp potentials are shown from these recordings. The holding potential for the recordings was −60 mV. The voltage-clamp protocol used for recording the traces can be seen under the traces. The duration of the voltage steps was 2 s, followed by a 10 s waiting time. During each voltage step at *t* = 500 ms, glycine was applied focally to the cell to elicit GlyR currents. **(B)** I-V-relations of the GlyR current traces shown in **(A)**. The GlyR-mediated currents have been plotted against the clamped membrane potentials shown in the inset of **(A)**. In the figure, *V*_m_ axis is crossed at E_Gly_ = −69 mV for KCC2^WT^ and E_Gly_ = −56 mV for KCC2^R231H^. **(C)** Cell selection was done based on fluorescence and cell morphology. Cells with an intermediate fluorescence level and a distinct, healthy-appearing morphology with possible neurites were opted for. Clearly unhealthy (e.g., diffuse, swollen) cells or cells with clutter (e.g., residue of disintegrated cells or dust speckles) in the vicinity of their cellular membrane were omitted, as the cells were approached horizontally and any excess material in the vicinity of the cellular membrane would risk affecting the formation of the giga-seal or clogging the patch pipette. **(D)** Results of the gramicidin-perforated patch-clamp recordings for N2a cells expressing KCC2^WT^, KCC2^R231H^ and mCherry (mock) visualized as box plots with also the data point distribution shown. E_Gly_ was determined from the recorded GlyR-mediated currents, and the determined reversal potentials were corrected for the LJP = 4.5 mV. For KCC2^WT^, median was determined to be −79.5 mV (25–75 interquartile ranges: −84, −69.75 mV), *n* = 13; for the variant KCC2^R231H^ −58.5 mV (−61.75, −54.25 mV), *n* = 10; and for mock-transfected cells −49.5 mV (−61, −44 mV), *n* = 16. *N* = 27, 1 to 3 cells per experiment. Asterisks depict significance levels according to Dunn's *post hoc* test to Kruskal–Wallis analysis of variance.

In the gramicidin-perforated patch-clamp experiments, the α1-GlyR forms ionotropic receptor channels in the plasma membrane of transfected cells. In response to binding glycine, the receptor channel opens, allowing Cl^−^ to permeate and flow through the membrane. Cl^−^-mediated GlyR currents were thus recorded in the voltage-clamp mode, and the E_Gly_ was determined from current-voltage relations derived from the clamped voltages and peak GlyR current amplitudes (Figures 2A–D). E_Gly_ was, expectedly, much more hyperpolarized in the cells expressing KCC2^WT^ than in the mock-transfected cells not expressing KCC2 (Figure 2D, see figure legend for values). The cells expressing the variant KCC2^R231H^ showed consistently more depolarized E_Gly_ levels than the wild-type counterparts (median values −58.5 and −79.5 mV, respectively, *p* = 0.0329) and tendency toward more hyperpolarized values than the mock-transfected cells (Figure 2D). However, the difference between the variant KCC2^R231H^ and mock-transfected cells was not statistically significant (*p* = 0.0575), possibly due to small sample sizes and the inherent variation deriving from the method of choice. This intermediate range indicates that while the KCC2^R231H^ still retains some Cl^−^ extrusion capability, it is clearly weakened in comparison to KCC2^WT^. The observed effect can be due to either the variant directly affecting the ion translocation function of the protein, reduced presence in the plasma membrane, or due to a combination of these mechanisms.

**Figure 3:**
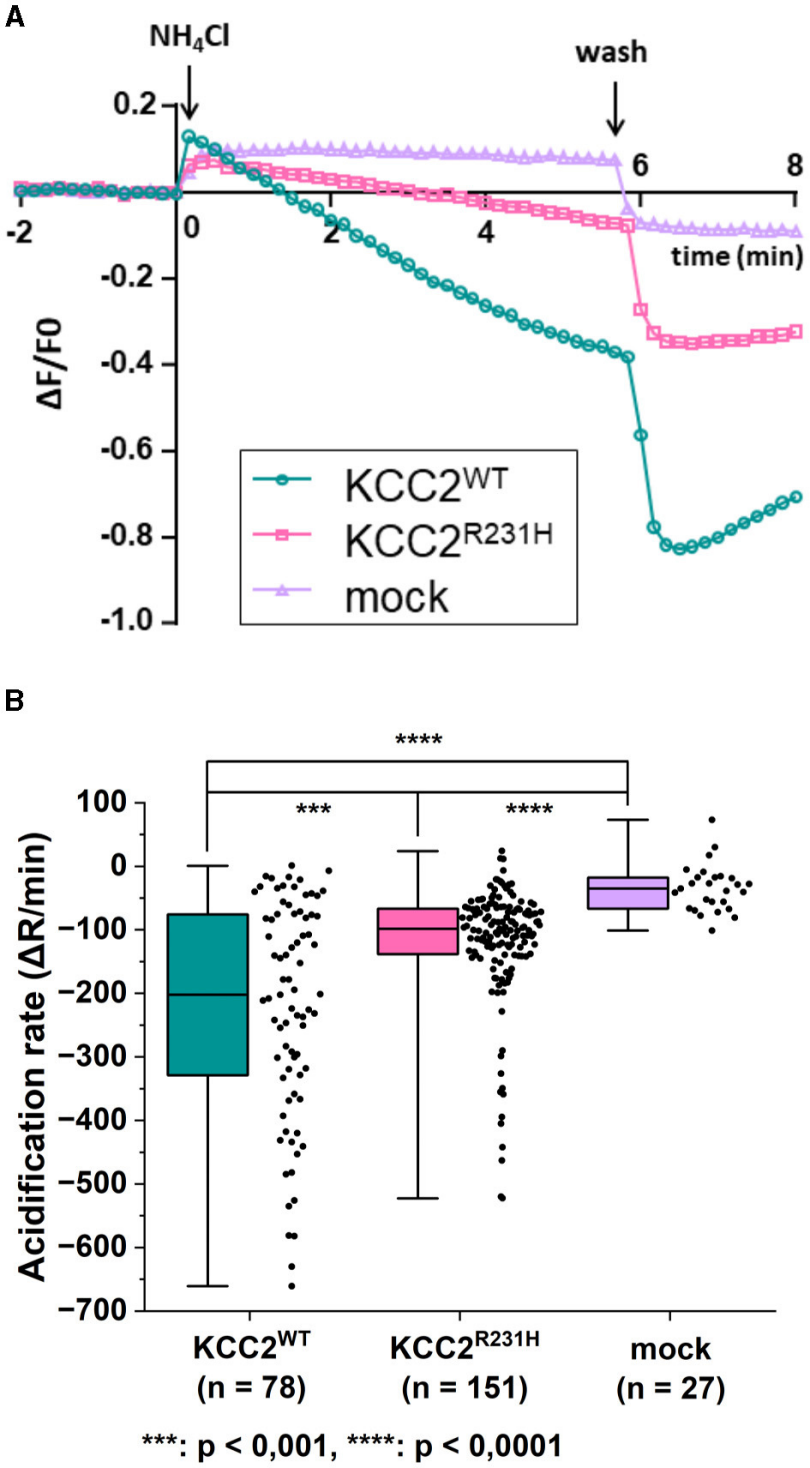
${\text{NH}}_{4}^{+}$ flux assay on N2a cells transiently co-transfected with either KCC2^WT^, KCC2^R231H^ or mCherry and pHluorin. **(A)** N2a cells transiently co-transfected with either KCC2^WT^, KCC2^R231H^ or mCherry and pH-sensitive pHluorin were perfused with 10 mM NH_4_Cl containing solution at *t* = 0. Swiftly after NH_4_Cl application, the intracellular space alkalizes as NH_3_ diffuses through the plasma membrane. In the KCC2 expressing cells, KCC2 begins to transport ${\text{NH}}_{4}^{+}$ into the cell, acidifying the intracellular space. The rate of pH_i_ change during ${\text{NH}}_{4}^{+}$ transportation, visualized by the fluorescence intensity of pHluorin, is dependent on the ion-transportation activity of KCC2. **(B)** The acidification rate of N2a cells expressing KCC2^WT^, KCC2^R231H^ or mCherry (mock) in conjunction with pHluorin visualized as box plots with the data point distribution shown. KCC2^R231H^ shows clear reduction in the acidification rates, indicating reduced ${\text{NH}}_{4}^{+}$ translocation across plasma membrane. The median value of rate of acidification of KCC2^WT^ transfected cells was determined to be −201.89 (−329.97, −75.54), *n* = 78; for KCC2^R231H^ transfected cells −98.52 (−138.04, −66.72), *n* = 151; and for mock-transfected cells −35.18 (−66.66, −17.78), *n* = 27. For KCC2^WT^ and mock, *p* = 1.6·10^−12^; for KCC2^WT^ and KCC2^R231H^, *p* = 0.00067; and for mock and KCC2^R231H^, *p* = 4.5·10^−7^. *N* = 12. Asterisks depict significance levels between groups according to Mann–Whitney *U*-test.

The results of the ${\text{NH}}_{4}^{+}$ flux assay were in line with those of the gramicidin-perforated patch-clamp experiments. In the ${\text{NH}}_{4}^{+}$ flux assay, the functionality of the variant protein was assessed by studying changes in the intracellular pH (pH_i_) in response to applications of ${\text{NH}}_{4}^{+}$ that serves as a surrogate ion for KCC2-dependent K^+^ flux (Payne, 1997; Hershfinkel et al., 2009). To measure the dynamics of pH_i_, we employed a pH-sensitive variant of GFP, pHluorin (Miesenböck et al., 1998). The application of 10 mM ${\text{NH}}_{4}^{+}$ to mock transfected cells produced a rapid alkalinization of the cytoplasm that persisted or slightly decreased during the presence of ${\text{NH}}_{4}^{+}$ in the recording saline (Figure 3A). Consistent with previous reports (Payne, 1997; Hershfinkel et al., 2009), the application of the same ${\text{NH}}_{4}^{+}$ containing media to cells expressing KCC2^WT^ resulted in progressive acidification of the pH_i_ after a slight and brief (10–30 s) alkalinization event (Figure 3A). The median values of rates of acidification for KCC2^WT^ and mock transfected cells were significantly different (Figure 3B, see figure legend for values). The cells expressing KCC2^R231H^ were characterized similarly to KCC2^WT^ by the presence of the acidification component during exposure to ${\text{NH}}_{4}^{+}$ (Figure 3A), but the overall rates of acidification were in intermediate position between KCC2^WT^ and mock-transfected cells (Figure 3B; median values of the rates of acidification for KCC2^WT^, KCC2^R231H^ and mock-transfected cells were −201.89, −98.52, and −35.18, respectively). The pairwise differences observed between all three groups were statistically significant (Figure 3B; see figure legend for *p*-values) and suggest reduced ion transport activity in KCC2^R231H^-expressing cells.

### 3.5 Live-cell surface immunolabeling

**Figure 4:**
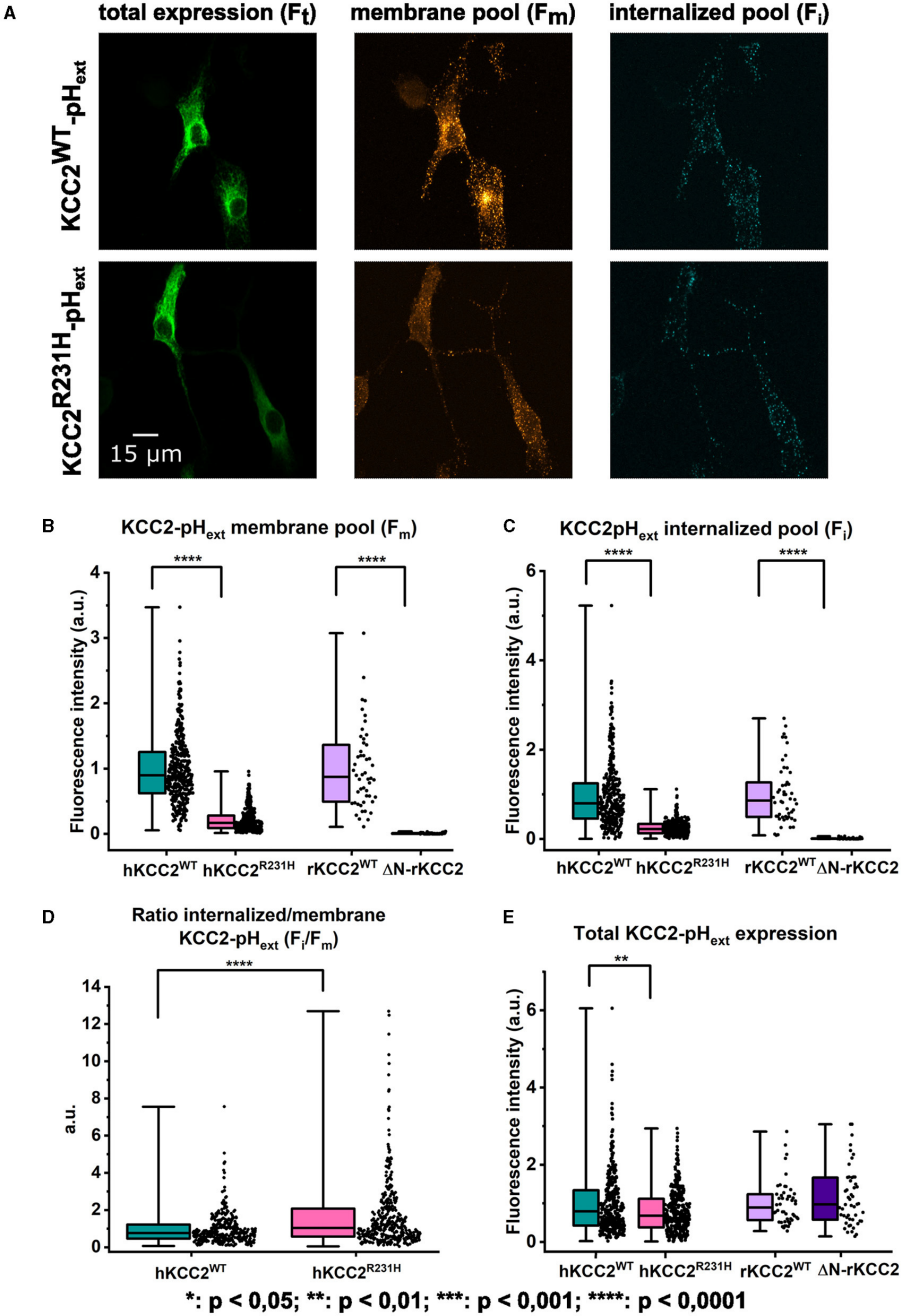
Cell surface expression of KCC2^WT^ and KCC2^R231H^ harboring the extracellular pHluorin-tag. **(B–E)** illustrate the quantitative analyses of the fluorescence emitted by different pools of mentioned surface labeled KCC2-pH_ext_ constructs. Two groups of transfected N2a cells were analyzed: KCC2^WT^-pH_ext_ vs. KCC2^R231H^-pH_ext_ (*N* = 14, *n* = 360 for KCC2^WT^-pH_ext_ and *n* = 368 for KCC2^R231H^-pH_ext_) and rKCC2^WT^-pH_ext_ vs. ΔN-rKCC2-pH_ext_ (*N* = 6, *n* = 50 for both rKCC2^WT^-pH_ext_ and ΔN-rKCC2-pH_ext_). The cumulative fluorescence per cell of each pool was normalized to the mean fluorescence of KCC2^WT^-pH_ext_ or rKCC2^WT^-pH_ext_ in each set of experiments. Asterisks depict significance values between KCC2^WT^-pH_ext_ and KCC2^R231H^-pH_ext_ and between rKCC2^WT^-pH_ext_ and ΔN-rKCC2-pH_ext_. Mann–Whitney *U*-test was utilized to determine significance levels between groups. **(A)** Total, membrane-bound and internalized pools of KCC2-pH_ext_ visualized as described in Methods. 12 *z*-planes acquired for each channel are composed together in single images. **(B)** The membrane-bound pools (*F*_m_). hKCC2^WT^-pH_ext_: 0.898 (0.623, 1.255); hKCC2^R231H^-pH_ext_: 0.170 (0.088, 0.280); rKCC2^WT^-pH_ext_: 0.873 (0.492, 1.365); ΔN-rKCC2-pH_ext_: 0.004 (0.001, 0.012). **(C)** Internalized pools (*F*_i_). hKCC2^WT^-pH_ext_: 0.797 (0.455, 1.244); hKCC2^R231H^-pH_ext_: 0.222 (0.127, 0.335); rKCC2^WT^-pH_ext_: 0.860 (0.492, 1.268); ΔN-rKCC2-pH_ext_: 0.006 (0.002, 0.012). **(D)** Internalized to membrane-bound pools ratio (*F*_i_/*F*_m_) quantified for each single cell. hKCC2^WT^-pH_ext_: 0.768 (0.459, 1.217); hKCC2^R231H^-pH_ext_: 1.034 (0.573, 2.083). **(E)** The fluorescence of total amount of KCC2-pH_ext_ constructs per cell (*F*_t_). hKCC2^WT^-pH_ext_: 0.795 (0.424, 1.341); hKCC2^R231H^-pH_ext_: 0.680 (0.379, 1.120); rKCC2^WT^-pH_ext_: 0.892 (0.565, 1.238); ΔN-rKCC2-pH_ext_: 0.974 (0.575, 1.670). For hKCC2^WT^-pH_ext_ and hKCC2^R231H^-pH_ext_ *p* = 0.009; for rKCC2^WT^-pH_ext_ and ΔN-rKCC2-pH_ext_ *p* = 0.431.

To investigate the effects of the variant on KCC2 membrane surface expression, we used the advantage of previously described (Friedel et al., 2017; Dumon et al., 2018) live-cell immunolabeling protocol allowing the visualization in N2a cells of the surface expressed and internalized molecules of KCC2-pH_ext_, a chimera protein composed of KCC2 and a pHluorin-tag inserted within the extracellular loop between transmembrane segments (TM) 3 and 4. The fluorescence intensity of three different pools of KCC2^WT^-pH_ext_ and KCC2^R231H^-pH_ext_ was determined: *F*_m_, molecules present on the cell surface of a single cell at the given time point; *F*_i_, surface labeled molecules internalized during a 1 h period and *F*_t_, the total amount of KCC2-pH_ext_ expressed into a single cell (see representative images on *F*_m_, *F*_i_, and *F*_t_ in Figure 4A). The specificity of the labeling of cell surface expressed molecules was verified in parallel experiments involving N2a cells transfected with rKCC2^WT^-pH_ext_ and ΔN-rKCC2-pH_ext_, a construct with a deleted N-terminus not attending the cell surface (Friedel et al., 2017). The values of *F*_m_ and *F*_i_ collected from cells expressing ΔN-rKCC2-pH_ext_ were more than 10-fold lower than those of KCC2-pH_ext_ expressing cells (Figures 4B, C) further confirming the inability of ΔN-rKCC2-pH_ext_ to reach the cell surface and validating the efficacy of the live-cell immunolabeling of cells expressing constructs harboring the pHluorin-tag. In terms of the human KCC2-pH_ext_ constructs, *F*_m_ and *F*_i_ were 5- and 3.6-fold higher in KCC2^WT^-pH_ext_ transfected cells than in their KCC2^R231H^-pH_ext_ counterparts, respectively (Figures 4B, C, see figure legends for the values). The ratio of internalized to surface labeled fluorescence units was, in turn, statistically significantly increased in cells expressing the KCC2^R231H^-pH_ext_ variant (Figure 4D). While the total quantities of overexpressed rKCC2^WT^-pH_ext_ and ΔN-rKCC2-pH_ext_ molecules, as revealed by *post-hoc* staining of fixed and permeabilized N2a cells, were similar, the total quantities for the variant KCC2^R231H^-pH_ext_ molecules in single cells were slightly, yet significantly lower compared to KCC2^WT^-pH_ext_ (median values for KCC2^R231H^-pH_ext_ and KCC2^WT^-pH_ext_, respectively, 0.680 and 0.795, *p* = 0.009; Figure 4E).

## 4 Discussion

The main findings of this study are:

(i) The identification of a rare new case of neurodevelopmental disorder associated with a previously unknown homozygous variant of *SLC12A5*.
(ii) The observation of a striking similarity of clinical phenotype with other previously reported cases of variants in the same gene *SLC12A5*; one homozygous variant with two described patients, and five cases of monogenic heterozygous composite variants.
(iii) Findings of the reduced ion-transport activity and decreased surface expression of the identified variant overexpressed *in vitro*.

**Figure 5:**
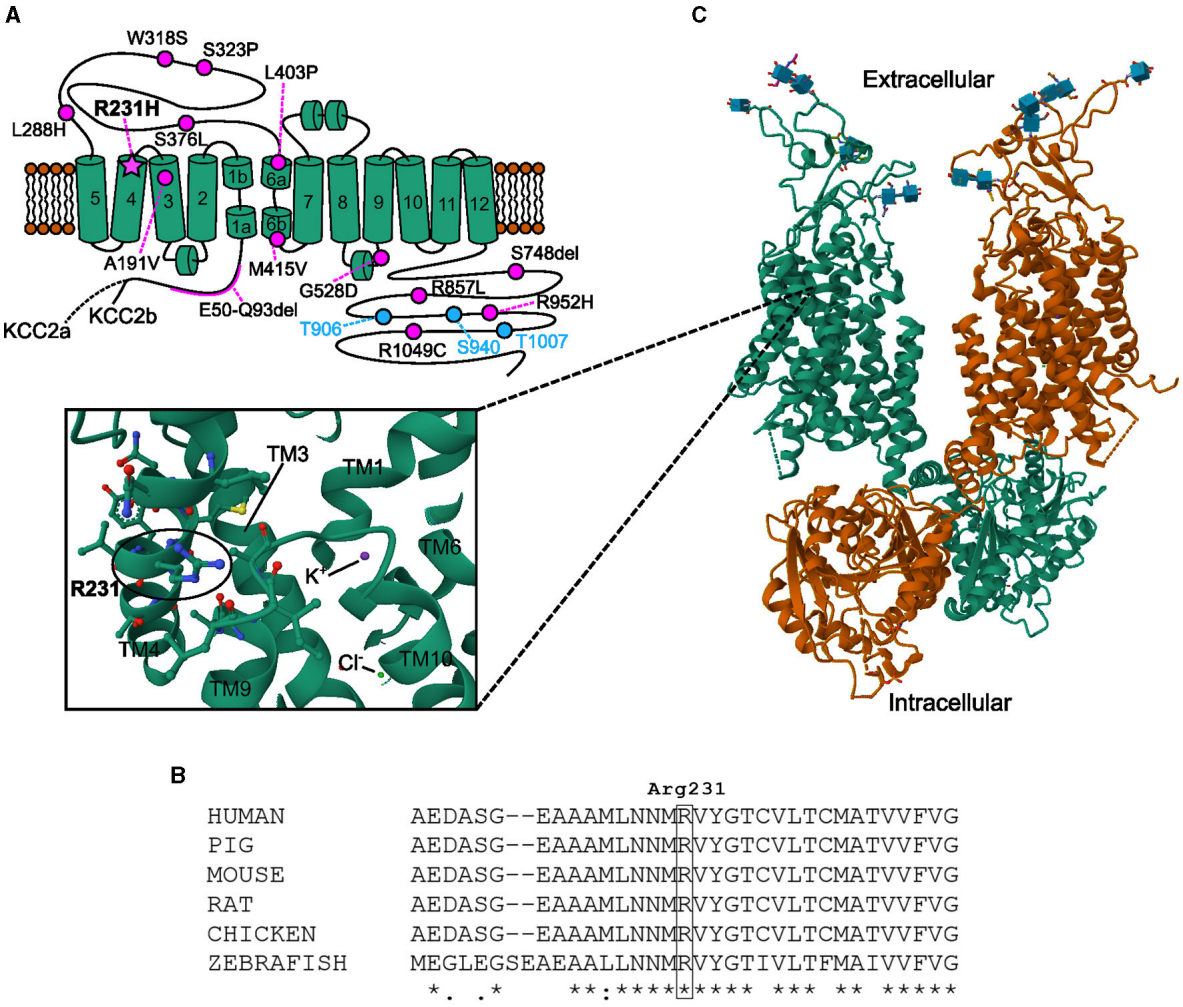
Membrane topology and structural modeling of the KCC2 protein with relevant sites and previously described variants highlighted. **(A)** Membrane topology of human KCC2 (KCC2a and KCC2b). Previously described disease-associated variant sites (Kahle et al., 2014; Puskarjov et al., 2014; Stödberg et al., 2015; Saitsu et al., 2016; Saito et al., 2017) are marked as pink circles along with residue numbering according to KCC2b. All variants are compound heterozygous except for the homozygous L288H associated with EIMFS (Stödberg et al., 2015) and R231H reported in this study (pink star). Blue circles depict residues critical for the phospho-regulation of KCC2 membrane trafficking and function. **(B)** The arginine residue 231 and its surrounding sequence are conserved among different species. Alignment of the amino acid sequences of human (Q9H2X9-2), pig (A0A4X1U489_PIG), mouse (S12A5_MOUSE), rat (S12A5_RAT), chicken (A0A8V0YI71_CHICK) and zebrafish (M1EVR1_DANRE) KCC2 was done with Clustal Omega (Sievers and Higgins, 2018). **(C)** Structural model of KCC2. KCC2 forms dimers with domain-swapping organization whereby the C-terminal of one monomer resides beneath the transmembrane domain of the other monomer. The sticks with blue cubes depict carbohydrate chains located in the large extracellular loop between TMs 5 and 6. The inset shows a zoomed view on the location of residue R231 (highlighted with a circle) within TM4, close to TMs 3, 9, and 10. The binding sites for K^+^ and Cl^−^ have also been indicated. The model images were created using Mol* Viewer (Sehnal et al., 2021) according to the KCC2 cryo-EM structure published by Chi et al. (2021). This structure can be found from the Research Collaboratory for Structural Bioinformatics Protein Data Bank (Berman et al., 2000) under the identification code (PDB ID) 6M23.

Of the so far described *SLC12A5* variants associated with EIMFS and undergone functional studies (Figure 5A), only one is a homozygous missense variant (L288H, two patients; Stödberg et al., 2015) while the rest of the variants are compound heterozygous (Kahle et al., 2014; Puskarjov et al., 2014; Stödberg et al., 2015; Saitsu et al., 2016; Saito et al., 2017). None of these variants reside within TM4, thus no disease associated variants in this region have altogether been described. The homozygous missense variant L288H, sitting in the extracellular domain (ECD) close to TM5, described by Stödberg et al. (2015), was determined to significantly decrease membrane expression with some retained functionality for the variant KCC2^L288H^. Our results point to a similar effect: the functionality of the variant KCC2^R231H^ is significantly decreased, but some functionality remains (Figures 2D, 3A, B), and the underlying cause for the decreased functionality seems to be, at least partly, decreased membrane trafficking (Figures 4B–D). Due to the homozygosity, there can be no compensation from a healthy wild-type allele, making the net effect on overall KCC2 functionality more sensitive for the type and location of the variant. Nevertheless, the geno-phenotype correlations between previously reported cases of *SLC12A5* variants and the one reported here clearly overlap and underline the importance and sensitivity of KCC2 in the onset of early infantile epilepsies and neurodevelopmental disorders.

Here, the functionality and expression of the novel *SLC12A5* missense variant R231H we discovered in a patient diagnosed with EIMFS and profound intellectual disability were assessed *in vitro* by gramicidin-perforated patch-clamp, ${\text{NH}}_{4}^{+}$ assay and surface immunolabeling. The results of the gramicidin-perforated patch-clamp (Figure 2D) show a significant depolarizing shift in E_Gly_, indicative of an increase in [Cl^−^]_in_ for the KCC2^R231H^ expressing cells and suggesting a weakened Cl^−^ extrusion capability for the variant protein. These results were corroborated by the ${\text{NH}}_{4}^{+}$ flux assay results (Figure 3B) where a significant decrease in the acidification rate of the KCC2^R231H^ expressing cells suggest a marked reduction in the ion-transport function of the co-transporter. Finally, in the surface immunolabeling experiments, the amount of KCC2^R231H^-pH_ext_ expressed in the plasma membrane during the tested time point was strongly decreased (Figure 4B). This finding suggests that the decreased surface expression of the KCC2 contributes to reduced ion-transport activity of the transporter and thus to reduced efficacy of [Cl^−^]_in_ maintenance. The decreased surface expression of KCC2^R231H^-pH_ext_ could be a result of a decreased rate of delivery of the protein to the plasma membrane or of enhanced protein internalization. Although the quantity of internalized KCC2^R231H^-pH_ext_ molecules was decreased in comparison to KCC2^WT^-pH_ext_ (Figure 4C), the relative amount of the internalized KCC2^R231H^-pH_ext_ pool (ratio *F*_i_/*F*_m_) was significantly higher than that of KCC2^WT^-pH_ext_ (Figure 4D). This increased internalization ratio could potentially explain the reduction in both the surface expression and ion transport activity of KCC2^R231H^-pH_ext_ variant. However, this explanation seems to be in conflict with the observation of the decreased size of the internalized pool of KCC2^R231H^-pH_ext_ in single cells. A recent study (Kok et al., 2024) described that KCC2, when overexpressed in heterologous expression systems, is targeted for rapid endoplasmic reticulum-associated degradation (ERAD). Therefore, a plausible explanation is that KCC2^R231H^ undergoes ERAD more efficiently than the wild-type counterpart, which aligns well with the finding of the reduced total amount of KCC2^R231H^ overexpressed in single N2a cells (Figure 4E). Interestingly Kok et al. (2024) report that ERAD of KCC2 was detected in heterologous expression systems but not in neurons. Future investigations involving analysis of the properties of KCC2^R231H^ endogenously expressed in neuronal cellular environments are necessary for understanding the exact mechanisms of the KCC2^R231H^ variant downregulation.

*SLC12A5* contains several evolutionarily conserved regions, the part of the TM4 encompassing the site of the variant being one (analysis performed with Aminode webtool http://aminode.org; Chang et al., 2018), with R231 itself being conserved across species (Figure 5B, analysis conducted utilizing Clustal Omega; Sievers and Higgins, 2018). *SLC12A5* is also among the 4.5% of genes that are most intolerant to genic variation (Petrovski et al., 2013). As stated in the results above, *in silico* analyses predicted the R231H variant to be probably damaging to protein function or structure, which is in line with the conservation of the residue and the site of the variant being evolutionarily conserved. However, despite the structures of the human KCCs of the *SLC12* gene family being fairly well-described (Xie et al., 2020; Chi et al., 2021), little is still known about the functionality of TM4, let alone the role of the R231 residue, especially in regard to KCC2 surface expression. It is, although, well-known that at least the C- and N-terminals are important for KCC2 membrane trafficking. The C-terminal domain (CTD) of the KCC2 protein is the site of several continuous posttranslational regulatory mechanisms, as the residues involved in phosphorylation and dephosphorylation, T906, S940, and T1007, are all located in the CTD. E.g., plasmalemmal trafficking and stability and, therefore, also Cl^−^ extrusion efficiency can be rapidly and reversibly regulated via phosphorylation and dephosphorylation of these sites (Kahle et al., 2013), and the CTD also plays a role in endocytosis (Zhao et al., 2008). The CTD is also important for the non-canonical functions of KCC2; it regulates neocortical developmental apoptosis (Mavrovic et al., 2020), mediates interactions through which KCC2 regulates the actin cytoskeleton in dendritic spines, and affects spinogenesis and spine morphogenesis (Li et al., 2007; Fiumelli et al., 2013; Awad et al., 2018). As the R231H substitution is located quite far from the CTD, close to the extracellular face of TM4 (Figures 5A, C), it seems unlikely that the transport-independent non-canonical functions or (de)phosphorylation mediated by the CTD would be affected by the substitution. The N-terminal domain (NTD) also partakes in posttranslational regulation, and according to Friedel et al. (2017), it is crucial in delivering KCC2 to the plasma membrane, whereas CTD mainly affects membrane stability of KCC2. Yet again, R231 does not reside close to either of these sites or residues, and without separate structural studies it is impossible to make inferences about any possible indirect interactions. Nevertheless, deducing from the surface immunolabeling experiment results and the significantly weakened pools of KCC2^R231H^-pH_ext_ labeled on the plasma membrane (Figures 4B–D), R231 and its surroundings still seem to be essential for membrane delivery through a yet unknown mechanism. One possibility is that the R231 residue may play a stabilizing role in membrane-protein dynamics, and its substitution by histidine could render the KCC2 protein chemistry less favorable for phospholipid membrane interactions.

This study also has certain methodological limitations, underlining the necessity for careful interpretation of results. Firstly, however powerful in determining the [Cl^−^]_in_ of single cells, gramicidin-perforated patch-clamp results can be influenced by several unwanted factors. These include the interference with cytosolic concentration of K^+^, effects of high input resistances on voltage-clamp quality, and the possibility for different holding potentials affecting the kinetics of Cl^−^ currents, and, thus, also the [Cl^−^]_in_. Secondly, in the ${\text{NH}}_{4}^{+}$ flux assay, the complexity of mechanisms affecting changes in the internal pH can cause significant variability in the observed fluorescence signals (Medina and Pisella, 2020). Despite their caveats, when combined, these methods nevertheless provide a strong means for assessing the functionality of KCC2, albeit, in possible future studies on the functionality of the variant, additional complementary methods should be considered. As briefly discussed above, significant limitations stem from the use of heterologous expression in cultured cells. This approach can be a good starting point for functional investigations and even necessary for structural biology but lacks power in explaining the significance of endogenous proteins in a more native and systemic setting. It becomes apparent that future *in vitro* studies on KCC2 variants should utilize isolated primary neurons or cortical neurons derived from induced pluripotent stem cells (iPSCs) as model systems. This is also necessary from the standpoint of researching the potential of KCC2 as a drug target. All in all, the role of KCC2 in the physiological setting is surprisingly extensive and not even restricted to the function of the brain. For example, the downregulation of KCC2 and the resulting dysfunctionality of the inhibitory system in the spinal cord may influence the genesis of neuropathic pain (Kitayama, 2018), and, interestingly, KCC2 is a viable potential biomarker for certain cancers (Wei et al., 2011; Chen et al., 2023).

To conclude, this study reinforces the association between *SLC12A5* pathogenic missense variants and EIMFS while also highlighting the importance of KCC2 in regulating the balance of normal neuronal activity. Based on our findings we cannot rule out possible direct effects of R231H on ion translocation function or protein degradation due to, e.g., possible protein misfolding due to the substitution. Our results do, however, clearly suggest that in KCC2^R231H^ the weakened membrane delivery leads to cytosolic Cl^−^ accumulation and hyperexcitation brought upon by impaired GABAergic inhibition, increasing the probability for epileptiform activity. As KCC2 activity seems to be diminished due to weakened surface expression, at least partially, it could be a suitable option to rescue Cl^−^ extrusion and the inhibitory effect of GABAergic signaling to alleviate seizure susceptibility by potentiating the functionality of the KCC2 present on the plasma membrane by pharmacological means, similar to as shown by Moore et al. (2018). KCC2 functionality can be potentiated by utilizing the multiple posttranslational regulatory pathways as described by Hartmann and Nothwang (2022). Although a promising prospect, so far it has been challenging to find suitable pharmacological compounds with high enough potency, efficacy and specificity and described KCC2 potentiation mechanisms (Prael et al., 2022). Nevertheless, since Cl^−^ homeostasis is such an essential part of the balance between neuronal excitation and inhibition, *SLC12A5* holds significant clinical potential, serving as both a therapeutic target and as a gene of risk due to susceptibility for pathogenic variants in epilepsies and epileptic encephalopathies.

## Data availability statement

The datasets presented in this study can be found in online repositories. The names of the repository/repositories and accession number(s) can be found at: www.fairdata.fi, https://doi.org/10.23729/2231c341-4e16-4576-8dd9-930ec98147c4; www.fairdata.fi, https://doi.org/10.23729/e7fa4fad-c947-49f5-8343-49681c7b5ff4; www.fairdata.fi, https://doi.org/10.23729/41614c78-1e44-45ea-bd95-5831fa7a3d57.

## Ethics statement

The studies involving humans were approved by Ethics Committee of the Northern Ostrobothnia Hospital District (EETTMK: 33/2014 and amendment 2021). The studies were conducted in accordance with the local legislation and institutional requirements. Written informed consent for participation in this study was provided by the participants' legal guardians/next of kin. Ethical approval was not required for the studies on animals in accordance with the local legislation and institutional requirements because only commercially available established cell lines were used. Written informed consent was obtained from the individual(s), and minor(s)' legal guardian/next of kin, for the publication of any potentially identifiable images or data included in this article.

## Author contributions

VJ: Formal analysis, Investigation, Methodology, Writing – original draft, Writing – review & editing. MHa: Formal analysis, Investigation, Methodology, Writing – review & editing. JK-E: Formal analysis, Investigation, Methodology, Writing – review & editing. ER: Formal analysis, Investigation, Methodology, Writing – review & editing. JP: Formal analysis, Investigation, Methodology, Writing – review & editing. MK: Formal analysis, Investigation, Methodology, Writing – review & editing. SMK: Methodology, Writing – review & editing. TN: Methodology, Writing – review & editing. MHu: Resources, Supervision, Writing – review & editing. RH: Resources, Supervision, Writing – review & editing. JU: Resources, Supervision, Writing – review & editing. IM: Methodology, Project administration, Resources, Supervision, Writing – review & editing. E-VI: Formal analysis, Investigation, Methodology, Project administration, Supervision, Writing – review & editing.
